# Variants in *ACPP* are associated with cerebrospinal fluid Prostatic Acid Phosphatase levels

**DOI:** 10.1186/s12864-016-2787-y

**Published:** 2016-06-29

**Authors:** Lyndsay A. Staley, Mark T. W. Ebbert, Daniel Bunker, Matthew Bailey, for the Alzheimer’s Disease Neuroimaging Initiative, Perry G. Ridge, Alison M. Goate, John S. K. Kauwe

**Affiliations:** Department of Biology, Brigham Young University, Provo, UT 84602 USA; Biology and Biomedical Sciences, Washington University, St. Louis, MO 63110 USA; Department of Neuroscience Icahn School of Medicine, New York, NY 10029 USA

**Keywords:** Brain, Cancer, CSF, PAP

## Abstract

**Background:**

Prostatic Acid Phosphatase (PAP) is an enzyme that is produced primarily in the prostate and functions as a cell growth regulator and potential tumor suppressor. Understanding the genetic regulation of this enzyme is important because PAP plays an important role in prostate cancer and is expressed in other tissues such as the brain.

**Methods:**

We tested association between 5.8 M SNPs and PAP levels in cerebrospinal fluid across 543 individuals in two datasets using linear regression. We then performed meta-analyses using METAL =with a significance threshold of *p* < 5 × 10^−8^ and removed SNPs where the direction of the effect was different between the two datasets, identifying 289 candidate SNPs that affect PAP cerebrospinal fluid levels. We analyzed each of these SNPs individually and prioritized SNPs that had biologically meaningful functional annotations in wANNOVAR (e.g. non-synonymous, stop gain, 3’ UTR, etc.) or had a RegulomeDB score less than 3.

**Results:**

Thirteen SNPs met our criteria, suggesting they are candidate causal alleles that underlie *ACPP* regulation and expression.

**Conclusions:**

Given PAP’s expression in the brain and its role as a cell-growth regulator and tumor suppressor, our results have important implications in brain health such as cancer and other brain diseases including neurodegenerative diseases (e.g., Alzheimer’s disease and Parkinson’s disease) and mental health (e.g., anxiety, depression, and schizophrenia).

**Electronic supplementary material:**

The online version of this article (doi:10.1186/s12864-016-2787-y) contains supplementary material, which is available to authorized users.

## Background

Prostatic Acid Phosphatase (PAP)—an enzyme expressed by the Acid Phosphatase, Prostate (*ACPP*) gene—is predominantly produced in the prostate, and is an important biomarker used to assess and monitor prostate cancer [1–3], but is also expressed in other tissues like the brain [4–7]. Recent research suggests PAP is a key cell growth regulator and potential tumor suppressor gene [8–10]. Additional studies demonstrate *ACPP* is expressed in the brain and suggest that PAP plays a critical role in preventing cell proliferation, cancer cell invasion, and neurite retraction [11, 12]. PAP’s function has critical implications in mental health diseases such as anxiety, depression, and schizophrenia [5], neurodegenerative diseases such as Alzheimer’s and Parkinson’s disease [13], and possibly in brain cancer. Yet, little is known about ACPP’s function and regulation in the brain.

To date, research regarding *ACPP* function is almost exclusively limited to prostate cancer research, but *ACPP* is known to be expressed at lower levels in other tissues, such as the brain [4–7]. Specifically, *ACPP* is expressed in brain regions related to language, motor coordination, cognitive function, and self-awareness [4–7], suggesting it plays an important role in brain health. While PAP’s role within nervous tissue is not fully elucidated, it is known to localize in synaptic nerve endings [5] and co-localizes with SNAPIN [14], a protein that binds to SNAP-25 [15], which is associated with schizophrenia [16–19]. Recent studies further suggest PAP is a key cell growth regulator and potential tumor suppressor gene in the prostate [8–10], but PAP may have similar roles in the brain. A study by Tanaka et al. demonstrated that PAP degrades lysophosphatidic acid (LPA) in seminal plasma [11], and LPA is known to stimulate cell proliferation and prevent apoptosis [11] and has also been strongly implicated in cancer cell invasion [12]. Tanaka et al. further reported that lysophosphatidylcholine (LPC) and lysophospholipase D (lysoPLD) are found in CSF [11]. LPC is an LPA precursor and lysoPLD produces LPA from LPC, suggesting that LPA is likely present in the brain. These data suggest PAP may play a significant role protecting the brain from *de novo* brain tumors and metastatic tumors by inhibiting cell proliferation and cancer cell invasion, respectively.

LPA is also known to cause neurite retraction [11]. Neurites are a general term for axons and dendrites, generally used when it’s not possible to differentiate between the two (e.g., during development). A corresponding study by Sayas et al. demonstrated that inducing differentiated SY-SH5Y human neuroblastoma cells with LPA causes neurite retraction and site-specific Alzheimer's disease-like Tau phosphorylation [13]. These studies further suggest PAP plays a critical role in neuronal health, perhaps especially during development.

A study by Nousiainen et al. explored PAP’s function in the brain using PAP knockout mice [5]. Their results demonstrate that PAP knockout mice have enlarged lateral ventricles, a common phenotype in movement and neurodegenerative disorders such as Alzheimer’s disease, dementias, bipolar disorder, schizophrenia, Parkinson’s disease, and Huntington’s disease [5]. They also observed increased anxiety in the mice and decreased prepulsed inhibition. Molecular explanations for their observations may include increased GABAergic transmission and mislocalization of SNAPIN [5]. Increased GABAeric transmission inhibits neuronal excitability [20] while mislocalization of SNAPIN may affect neurotransmitter release [5], both of which may affect neuronal homeostasis and brain health.

Here we have conducted a genome-wide association study of PAP levels in cerebrospinal fluid (CSF) in 543 individuals from two datasets. Further characterization of the variants that we have identified may lead to a deeper understanding of PAP regulation and provide important insights into its effects on prostate cancer, brain cancer, mental health disorders, and neurodegenerative diseases.

## Methods

A. Subjects and data description

CSF samples were collected from 246 individuals in the Knight-Alzheimer’s Disease Research Center at Washington University School of Medicine (Knight ADRC) and from 297 individuals in the Alzheimer’s Disease Neuroimaging Initiative (ADNI). Alzheimer’s Disease status was 93.5 % control and 6.5 % case in the Knight ADRC samples, and 86.5 % and 13.5 % in the ADNI samples. Levels for 190 biomarkers were measured for each sample using the Human DiscoveryMAP Panel v1.0 and a Luminex 100 platform [21] and samples were genotyped using the Illumina 610 or the Omniexpress chip. A description of the Knight ADRC samples and the associated CSF collection methods has been previously published [22, 23]. ADNI CSF samples were collected as part of the ADNI biomarker study [24], and were obtained from the ADNI database (adni.loni.usc.edu). All samples are of European descent. All individuals whose data were included in this study were explicitly consented, following appropriate Institutional Review Board policies.

B. SNP imputation

SNPs were imputed as previously described. Briefly, data from the 1000 Genomes Project (June 2012 release) were used to impute SNPs using Beagle. Imputed SNPs with the following criteria were removed: (1) an r^2^ of 0.3 or lower, (2) a minor allele frequency (MAF) lower than 0.05 (3) out of Hardy-Weinberg equilibrium (*p* < 1 × 10 − 6), (4) a call rate lower than 95 %, or (5) a Gprobs score lower than 0.90. Exactly 5,815,690 SNPs passed the QC process.

C. Data cleaning and analysis

All analyses were conducted using PLINK, a whole genome association analysis toolset [25]. We performed genotype quality control on the Knight ADRC and ADNI CSF datasets by first excluding SNPs that exceeded thresholds for Hardy-Weinberg Equilibrium [26, 27] (−−hwe 0.00001), missing genotype rate (−−geno 0.05), and minor allele frequency (−−maf 0.01). We then excluded individuals with a missing genotyping rate greater than 2 % (−−mind 0.02). Exactly 246 individuals from Knight ADRC and 282 samples from ADNI remained after data cleaning. Remaining Knight ADRC and ADNI samples consisted of 40 % and 61 % males, respectively. ADNI samples varied in age from 58 to 91 years, with an average age of 76 years, and Knight ADRC samples varied in age from 49 to 91 years, with an average age of 73 years.

After data cleaning, we conducted a linear regression for all remaining SNPs within each data set to test for an association with CSF PAP levels, adjusting for age, gender, and the first two principle components generated using EigenSoft [28, 29]. The full script for our PLINK analyses is found in the Appendix. We then performed a meta-analysis across both data sets, accounting for sample size, p-values, and direction of effect using the default METAL [30] settings. We have included our scripts in Additional files 1, 2, 3, 4, 5, 6 and 7 for convenience.

We retained all SNPs that had a meta-analysis *p*-value less than 5 × 10^−8^ and that had the same direction of effect in both the Knight ADRC and ADNI datasets. We searched for all SNPs in the NHGRI catalog of published genome-wide association studies [31] (downloaded July 16^th^, 2015) for known disease associations. We then used RegulomeDB [32] and functional annotations from wANNOVAR [33, 34] to identify SNPs that are biologically likely to modify gene function or expression. Specifically, we retained the 10 most significant SNPs and all significant SNPs with a RegulomeDB score less than 3 or that were non-synonymous, stop-gain, splice-site modifying, etc., or are in untranslated regions (UTRs). UTR SNPs have been shown to modify gene transcription and translation [35, 36]. RegulomeDB scores range from “1a” to “6”. Lower scores indicate stronger evidence that the SNP affects gene regulation based on both empirical data, such as ChIP-seq, and whether the SNP is within a known transcription factor binding motif. Any RegulomeDB score between “1a” and “1f” indicates the SNP is within a known expression quantitative trait locus (eQTL), is known to have transcription factors bind, and may have additional evidence. While these scores indicate functional effects, the associated SNP is not necessarily the causal variant.

All SNPs that met our inclusion criteria and are in or near the given region were also reanalyzed using conditional analyses to test whether there is one or multiple independent effects in the region [37]. Conditional analysis is a follow-up method used to test if there are secondary association signals within a region by retesting each SNP while including the top SNP as a covariate. We chose the most significant SNP in the region to use as a covariate in the conditional analyses.

## Results

Our meta-analysis yielded 289 SNPs significantly associated with PAP CSF levels (Additional file 8). Of the 289 SNPs, 276 are located on chromosome 3, in or near the *ACPP* gene, which is the gene that codes for PAP. We generated plots in R failed to detect evidence of inflation (genomic inflation factor = 1; Additional files 9 and 10).

**Fig. 1:**
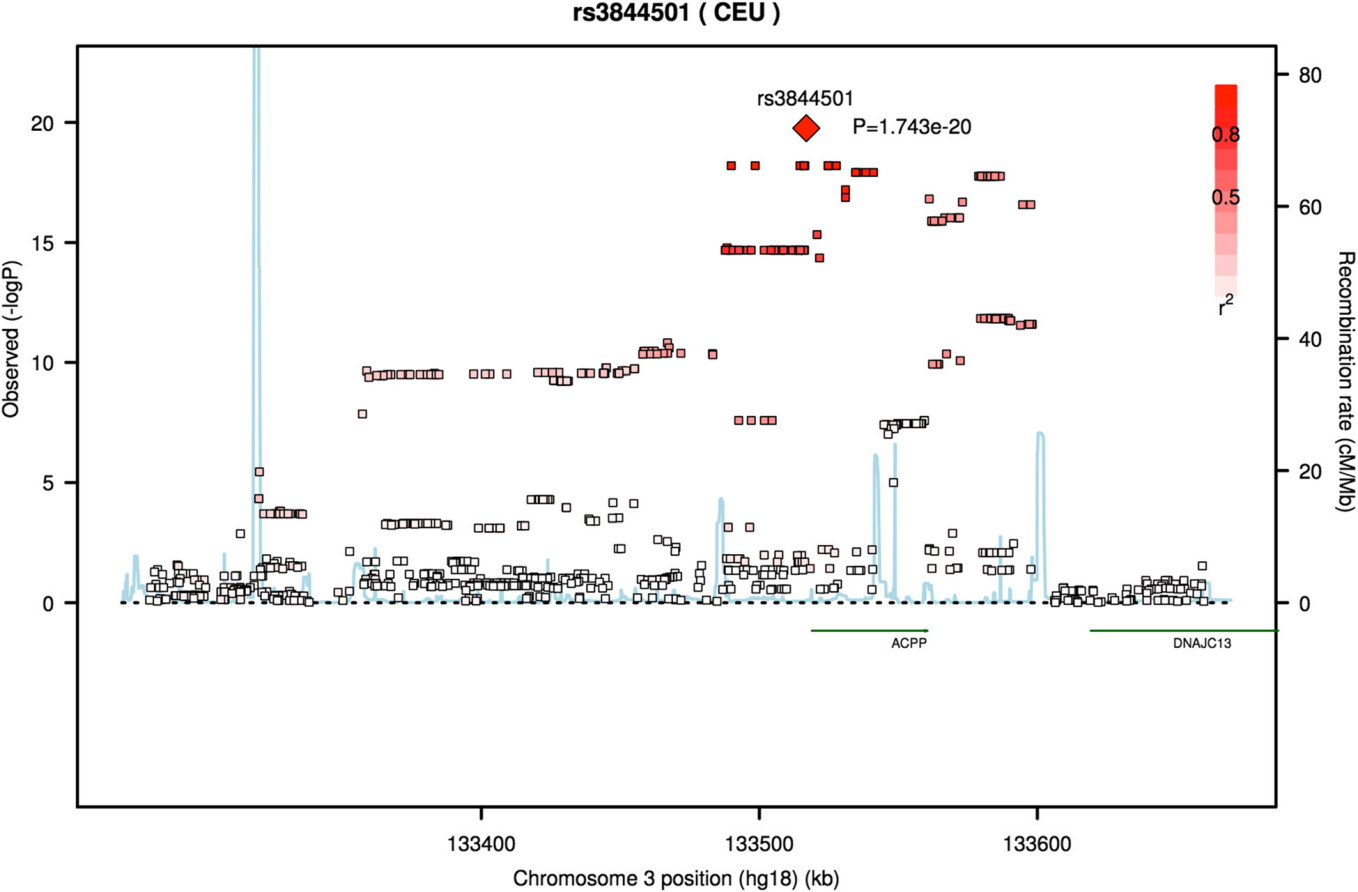
Regional association plot showing rs3844501 has the strongest association with PAP CSF levels. We identified several SNPs associated with PAP levels in CSF that are in or near the *ACPP* gene, which codes for PAP, and plotted association p-values in the region. rs3844501 has the strongest association with PAP CSF levels and is located approximately 2000 nucleotides upstream of the *ACPP* transcription start site. LD between rs3844501 and other SNPs in the region is strong

We explored potential causal SNPs and identified 23 that met our inclusion criteria, including the top 10 SNPs with the most significant p-values, and the 13 most biologically significant SNPs based on their RegulomeDB scores and functional annotations (Table 1). None of the 289 SNPs were associated with human disease in the NHGRI GWAS catalog. Two SNPs, rs16839055 and rs17182812, both received the score of “1f” from RegulomeDB. In this case, both SNPs are in LD with SNPs known to be associated with ACPP expression [31]. SNP rs3844501 has the strongest association with PAP CSF levels (*p* = 1.743e-20) and is approximately 2000 nucleotides upstream from the ACPP transcription start site (Fig. 1). Numerous SNPs proximal to rs3844501 are also highly associated and in strong LD. SNP rs3889987 has a RegulomeDB score of “2a”, implying it affects transcription factor binding because transcriptions factors have been observed binding to the SNP, the SNP is in a transcription factor motif, and matches a DNase footprint. There are 7 SNPs (rs11714139, rs56226080, rs73211958, rs56030168, rs4257547, rs11928839, and rs11917521) that received scores of 2b, showing they are also known to affect transcription factor binding and could affect PAP levels. The score of 2a is ranked higher than 2b, indicating rs3889987 is more likely to affect gene regulation. While 2a and 2b are similar, 2a is known to specifically match transcription factor motifs. SNPs, rs14192, rs1804136, and rs1042330 are all found in the 3’UTR, an important gene region for regulation.

**Table 1:** This table includes the 10 most significant SNPs and 13 other significant SNPs that have a RegulomeDB score less than 3, are non-synonymous, a splicing variant, or are located in untranslated regions (UTRs). All 23 SNPs are located in or near the ACPP gene on chromosome 3, which codes for PAP. There were no significant non-synonymous SNPs. The table includes SNP ids, reference and alternate alleles, minor allele frequency, predicted SNP function, and its directions in the Knight ADRC and ADNI data sets. Also included are the *p*-values from ADNI and Knight ADRC alone, their meta-analysis *p*-value and their RegulomeDB score, where available

SNP	Minor Allele	Major Allele	MAF	Predicted Function	Direction	ADNI *p*-value	Knight ADRC *p*-value	Meta-analysis *p*-value	RegulomeDB score
rs3844501	T	G	0.1458	Intergenic	++	1.972e-13	7.974e-09	1.743e-20	6
rs3762671	T	C	0.1060	Intergenic	++	3.815e-13	3.245e-08	1.515e-19	6
rs11716607	G	A	0.0923	Intronic	++	1.708e-12	3.615e-08	6.397e-19	No Data
rs56158166	T	G	0.0921	Intronic	++	1.708e-12	3.615e-08	6.397e-19	No Data
rs73213842	A	T	0.0923	Intergenic	++	1.708e-12	3.615e-08	6.397e-19	No Data
rs11706024	A	G	0.0921	Intergenic	++	1.708e-12	3.615e-08	6.397e-19	5
rs113143077	A	G	0.0923	Intergenic	++	1.708e-12	3.615e-08	6.397e-19	6
rs17344445	A	G	0.0923	Intergenic	++	1.708e-12	3.615e-08	6.397e-19	6
rs2887519	A	G	0.0923	Intergenic	++	1.708e-12	3.615e-08	6.397e-19	6
rs56073503	A	T	0.0921	Intronic	++	1.708e-12	3.615e-08	6.397e-19	6
rs3889987	T	G	0.0913	Intergenic	++	2.15E-13	4.08E-07	1.77E-18	2a
rs56030168	G	A	0.1769	5upstream	++	2.94E-11	1.34E-06	4.65E-16	2b
rs11714139	A	G	0.1232	Intergenic	++	1.25E-07	4.81E-04	5.41E-10	2b
rs56226080	C	G	0.1228	Intergenic	++	1.25E-07	4.81E-04	5.41E-10	2b
rs73211958	A	C	0.1218	Intergenic	++	1.25E-07	5.11E-4	5.82E-10	2b
rs16839055	T	C	0.3081	Intergenic	++	2.49E-05	2.52E-04	2.55E-08	1f
rs1804136	T	G	0.3962	UTR3	++	4.80E-05	1.39E-04	2.57E-08	No Data
rs14192	C	T	0.3964	UTR3	++	4.80E-05	1.39E-04	2.57E-08	5
rs1042330	A	G	0.3962	UTR3	++	6.49E-05	1.39E-04	3.43E-08	No Data
rs17182812	T	C	0.3962	Intronic	++	6.49E-05	1.39E-04	3.43E-08	1f
rs4257547	G	C	0.4113	Intronic	++	5.19E-05	1.94E-04	3.87E-08	2b
rs11928839	A	C	0.3954	Intronic	++	6.49E-05	1.60E-04	3.93E-08	2b
rs11917521	C	T	0.3954	Intronic	++	6.49E-05	1.60E-04	3.93E-08	2b

We performed conditional analyses including rs3844501 as a covariate in the linear regression. No SNPs in or near *ACPP* were significant, suggesting there is one association signal within the *ACPP* region.

## Discussion

We identified 289 SNPs that show replicable association with CSF PAP levels, the majority of which are found in or close to *ACPP*, the gene that codes for PAP. Among these, 23 SNPs in or near *ACPP* are highly significant and demonstrate evidence of functional effects, making them top candidates for being the causal allele for this association.

Conditional analyses strongly suggest there is a single signal within the region. As is the case with any association study, LD facilitates the discovery of associations, but makes identifying the causal allele difficult. The SNP most strongly associated with PAP CSF levels, rs3844501, is approximately 2000 nucleotides upstream of the *ACPP* transcription start site and has no known functional impact. Data from RegulomeDB identifies rs16839055 and rs17182812 as the two most likely candidate variants, however RegulomeDB is not exhaustive, and there are several other plausible candidates.

Based on current evidence, there are numerous mechanisms that can affect PAP protein levels in CSF including transcription, translation, feedback loops, and damaged proteins. Transcription is often most suspect in expression analyses and one of the top SNPs we identified, rs56030168, is most likely to affect transcription because it is less than 300 nucleotides upstream from the *ACPP* transcription start site and, according to the RegulomeDB score of “2b”, is known to have transcription factors bound at that location and is a known DNase peak. Given rs56030168’s location and other evidence, it may affect transcription factor binding. Rs16839055 is also upstream of *ACPP* and may affect transcription, but is more than 26,000 nucleotides upstream from the transcription start site.

The 3’UTR SNPs rs14192, rs1804136, and rs1042330 could affect PAP levels by affecting both transcription and translation. UTR regions are known to affect mRNA stability, transport, and translation processes [38–41]. Any of these three SNPs may play an important role in *ACPP* or PAP regulation.

None of the top 23 SNPs we identified were in *ACPP* exons, but several are intronic. Intronic variants can affect protein structure through splice modifications and have even been experimentally shown to affect transcription [42]. While intronic variants are typically less suspect, they may play an important role in PAP levels.

While these SNPs are the most significant and biologically likely eQTLs based on our criteria, there may be other candidate eQTLs in the list of 289. They all met the genome-wide significance p-value threshold and have matching effect directions, but more biological data will be necessary to support or refute their direct involvement. Additionally, our data are not whole exome or genome and there may be causal variants in LD with our top hits that were not genotyped. Full sequencing data within the region may reveal other candidate causal variations. Further research will be necessary to know which SNPs affect PAP CSF levels, and particularly whether they contribute to prostate cancer and other PAP-related functions and diseases.

## Conclusions

A plethora of studies suggest PAP plays an important role in prostate cancer, but recent studies suggest that PAP may play a critical role in brain health ranging from cancers to various brain disorders. Based on current research, we hypothesize that PAP’s role in brain health includes protecting against cancer development and metastasis, protecting against neuronal death by regulating LPA levels, and generally protecting brain health by contributing to neuronal homeostasis. In summary, this study has identified a clear and replicable QTL in *ACPP* for CSF PAP levels. Additional investigation of this locus may lead to a better understanding of *ACPP* regulation in the brain and additional insights into PAP’s role in the brain.

## Abbreviations

ACPP, Acid Phosphatase, Prostate; ADNI, Alzheimer’s Disease Neuroimaging Initiative; CSF, cerebrospinal fluid; eQTL, expression quantitative trait locus; Knight ADRC, Knight-Alzheimer’s Disease Research Center at Washington University School of Medicine; LPA, lysophosphatidic acid; LPC, lysophosphatidylcholine; lysoPLD, lysophospholipase D; MAF, minor allele frequency; PAP, Prostatic Acid Phosphatase; SNP, single nucleotide polymorphism; UTRs, untranslated regions.

## Additional files

Additional file 1: File contains the PLINK script used to clean data and find associations between SNPs and prolactin levels in the samples. (DOCX 101 kb) Additional file 2: File contains the forge_metal.py program, which is used to run METAL on the samples form each dataset to combine data. (DOCX 85 kb) Additional file 3: File includes the command for running forge_metal.py on ADNI dataset. (DOCX 36 kb) Additional file 4: File includes the command for running forge_metal.py on WU dataset. (DOCX 35 kb) Additional file 5: File include the contents of METAL meta analysis script. (DOCX 44 kb) Additional file 6: File include the command to run METAL software. (DOCX 29 kb) Additional file 7: File contains the command to sort combined METAL results. (DOCX 40 kb) Additional file 8: File contains a table of 289 Significant SNPs. (DOCX 124 kb) Additional file 9: File contains a Q-Q plot of the CSF in the ADNI samples. (DOCX 54 kb) Additional file 10: File contains a Q-Q plot of the CSF in the Knight ADRC samples. (DOCX 52 kb)
