# Supplementary material for: Variants in ACPP are associated with cerebrospinal fluid Prostatic Acid Phosphatase levels
Source: BMC Genomics. 2016 Jun 29;17(Suppl 3):439. doi: 10.1186/s12864-016-2787-y (PMC4943489; doi:10.1186/s12864-016-2787-y)
Supplement: Additional file 1: — File contains the PLINK script used to clean data and find associations between SNPs and prolactin levels in the samples. (DOCX 101 kb) [file 12864_2016_2787_MOESM1_ESM.docx]

Contents of plink script:

#!/usr/local/bin/bash

VERSION=4

PLINK_BIN=/path/to/plink/executable

SOURCE_DIR=/path/to/binary/and/covar/files

SOURCE_FILE=$SOURCE_DIR/bfile

DEST_DIR=/path/to/plink/results

mkdir -p $DEST_DIR

#parameters

HWE=0.00001

GENO=0.05

MIND=0.02

MAF=0.01

COVARS=age,gender,PC1All,PC2All

# PAP_CSF: WU

$PLINK_BIN --noweb --bfile $SOURCE_FILE --hwe $HWE --geno $GENO --maf $MAF --make-bed --out $DEST_DIR/PAP_CSF_WU_Geno_v$VERSION

$PLINK_BIN --noweb  --bfile $DEST_DIR/PAP_CSF_WU_Geno_v$VERSION --mind $MIND --make-bed --out $DEST_DIR/PAP_CSF_WU_Geno_MIND_v$VERSION

$PLINK_BIN --noweb --bfile $DEST_DIR/PAP_CSF_WU_Geno_MIND_v$VERSION --covar $SOURCE_DIR/covar.txt --covar-name $COVARS --pheno $SOURCE_DIR/wu_pheno_March2015.txt --pheno-name PAP_CSF --linear --adjust --out $DEST_DIR/PAP_CSF_WU_FINAL_v$VERSION

# PAP_CSF: ADNI

$PLINK_BIN --noweb --bfile $SOURCE_FILE --hwe $HWE --geno $GENO --maf $MAF --make-bed --out $DEST_DIR/PAP_CSF_ADNI_Geno_v$VERSION

$PLINK_BIN --noweb  --bfile $DEST_DIR/PAP_CSF_ADNI_Geno_v$VERSION --mind $MIND --make-bed --out $DEST_DIR/PAP_CSF_ADNI_Geno_MIND_v$VERSION

$PLINK_BIN --noweb --bfile $DEST_DIR/PAP_CSF_ADNI_Geno_MIND_v$VERSION --covar $SOURCE_DIR/covar.txt --covar-name $COVARS --pheno $SOURCE_DIR/adni_pheno_March2015.txt --pheno-name PAP_CSF --linear --adjust --out $DEST_DIR/PAP_CSF_ADNI_FINAL_v$VERSION
