# Supplementary material for: Variants in ACPP are associated with cerebrospinal fluid Prostatic Acid Phosphatase levels
Source: BMC Genomics. 2016 Jun 29;17(Suppl 3):439. doi: 10.1186/s12864-016-2787-y (PMC4943489; doi:10.1186/s12864-016-2787-y)
Supplement: Additional file 2: — File contains the forge_metal.py program, which is used to run METAL on the samples form each dataset to combine data. (DOCX 85 kb) [file 12864_2016_2787_MOESM2_ESM.docx]

Contents of forge_metal.py program:

import argparse

class Forger:

def __init__(self):

self.parse_args()

self.data = {}

def parse_files(self):

with open(self.args.bim_file, 'r') as bim_file:

for line in bim_file:

chrom,snp_rs,field_3,position,tested_allele,ref_allele = line.split()

self.data[snp_rs] = {'ref_allele': ref_allele, 'tested_allele': tested_allele}

with open(self.args.assoc_file, 'r') as assoc_file:

for line in assoc_file:

if 'ADD' in line:

chrom,snp_rs,position,tested_allele,test,nmiss,beta,stat,pvalue = line.split()

self.data[snp_rs]['beta'] = beta

self.data[snp_rs]['pvalue'] = pvalue

def output_file(self):

with open(self.args.out_file, 'w') as out_file:

out_file.write('%s\t%s\t%s\t%s\t%s\n' % ('SNP', 'RefAllele', 'NonRefAllele', 'P-value', 'Effect'))

for snp_rs in self.data.keys():

snp = self.data[snp_rs]

ref_allele = snp['ref_allele']

tested_allele = snp['tested_allele']

pvalue = snp['pvalue']

beta = snp['beta']

out_file.write('%s\t%s\t%s\t%s\t%s\n' % (snp_rs,ref_allele,tested_allele,pvalue,beta))

def parse_args(self):

parser = argparse.ArgumentParser(prog='Metal Forger', description='A script for combining plink output files into one METAL input file',

add_help=True)

parser.add_argument('--bim',

help='The .bim plink file containing the Tested allele and the Reference allele for each SNP',

type=str,

dest="bim_file")

parser.add_argument('--assoc',

help='The .assoc.linear plink file containing P value and BETA value',

type=str,

dest="assoc_file")

parser.add_argument('-o', '--out',

help='The output file to save results to',

type=str,

dest="out_file")

self.args = parser.parse_args()

if __name__ == '__main__':

f = Forger()

f.parse_files()

f.output_file()
