# Supplementary material for: Variants in ACPP are associated with cerebrospinal fluid Prostatic Acid Phosphatase levels
Source: BMC Genomics. 2016 Jun 29;17(Suppl 3):439. doi: 10.1186/s12864-016-2787-y (PMC4943489; doi:10.1186/s12864-016-2787-y)
Supplement: Additional file 3: — File includes the command for running forge_metal.py on ADNI dataset. (DOCX 36 kb) [file 12864_2016_2787_MOESM3_ESM.docx]

Command for running forge_metal.py on ADNI dataset: “python forge_metal.py --bim /path/to/plink/results/PAP_CSF_ADNI_results.bim --assoc /path/to/plink/results/PAP_CSF_ADNI_results.assoc.linear --out /path/to/plink/results/PAP_CSF_ADNI_Metal_input.txt”
