# Supplementary material for: Variants in ACPP are associated with cerebrospinal fluid Prostatic Acid Phosphatase levels
Source: BMC Genomics. 2016 Jun 29;17(Suppl 3):439. doi: 10.1186/s12864-016-2787-y (PMC4943489; doi:10.1186/s12864-016-2787-y)
Supplement: Additional file 5: — File include the contents of METAL meta analysis script. (DOCX 44 kb) [file 12864_2016_2787_MOESM5_ESM.docx]

Contents of METAL meta analysis script (named PAP_CSF_Metal_Script.txt):

MARKER  SNP

ALLELE  RefAllele NonRefAllele

PVALUE  P-value

EFFECT  Effect

#=========== Process Files ==========#

PROCESS /path/to/plink/results/PAP_CSF_ADNI_Metal_input.txt

PROCESS /path/to/plink/results/PAP_CSF_WU_Metal_input.txt

OUTFILE /path/to/plink/results/PAP_CSF_Meta_Analysis .txt

ANALYZE

QUIT
