# Supplementary material for: Variants in ACPP are associated with cerebrospinal fluid Prostatic Acid Phosphatase levels
Source: BMC Genomics. 2016 Jun 29;17(Suppl 3):439. doi: 10.1186/s12864-016-2787-y (PMC4943489; doi:10.1186/s12864-016-2787-y)
Supplement: Additional file 6: — File include the command to run METAL software. (DOCX 29 kb) [file 12864_2016_2787_MOESM6_ESM.docx]

Command to run METAL software: “./metal PAP_CSF_Metal_Script.txt”
