# Supplementary material for: Variants in ACPP are associated with cerebrospinal fluid Prostatic Acid Phosphatase levels
Source: BMC Genomics. 2016 Jun 29;17(Suppl 3):439. doi: 10.1186/s12864-016-2787-y (PMC4943489; doi:10.1186/s12864-016-2787-y)
Supplement: Additional file 7: — File contains the command to sort combined METAL results. (DOCX 40 kb) [file 12864_2016_2787_MOESM7_ESM.docx]

Command to sort combined METAL results: “sort -k6 -g /path/to/plink/results/PAP_CSF_Meta_Analysis1.txt > /path/to/plink/results/PAP_CSF_Meta_Analysis1.txt.sorted”

Note that “-k6” lets us use the 6th column as the sorting key, which is interpreted as a number (including scientific representations such as 1e^18^) with the “-g” option.
