# Supplementary material for: Variants in ACPP are associated with cerebrospinal fluid Prostatic Acid Phosphatase levels
Source: BMC Genomics. 2016 Jun 29;17(Suppl 3):439. doi: 10.1186/s12864-016-2787-y (PMC4943489; doi:10.1186/s12864-016-2787-y)
Supplement: Additional file 8: — File contains a table of 289 Significant SNPs. (DOCX 124 kb) [file 12864_2016_2787_MOESM8_ESM.docx]

| MarkerName | Allele  1 | Allele  2 | Weight | Zscore | P-value | Direction |
| --- | --- | --- | --- | --- | --- | --- |
| rs3844501 | a | c | 2 | 9.277 | 1.74E-20 | ++ |
| rs3762671 | t | c | 2 | 9.044 | 1.52E-19 | ++ |
| 3:132024143 | a | g | 2 | 8.885 | 6.40E-19 | ++ |
| rs11706024 | a | g | 2 | 8.885 | 6.40E-19 | ++ |
| rs11716607 | a | g | 2 | -8.885 | 6.40E-19 | -- |
| rs17344445 | a | g | 2 | 8.885 | 6.40E-19 | ++ |
| rs2887519 | a | g | 2 | 8.885 | 6.40E-19 | ++ |
| rs56073503 | a | t | 2 | 8.885 | 6.40E-19 | ++ |
| rs56158166 | t | g | 2 | 8.885 | 6.40E-19 | ++ |
| rs73213842 | a | t | 2 | 8.885 | 6.40E-19 | ++ |
| rs73213844 | a | c | 2 | -8.885 | 6.40E-19 | -- |
| rs113309163 | c | g | 2 | 8.813 | 1.22E-18 | ++ |
| rs11706011 | a | t | 2 | 8.813 | 1.22E-18 | ++ |
| rs56195646 | t | c | 2 | 8.813 | 1.22E-18 | ++ |
| rs73215917 | a | g | 2 | -8.813 | 1.22E-18 | -- |
| rs10935005 | a | g | 2 | -8.771 | 1.77E-18 | -- |
| rs11706944 | a | g | 2 | 8.771 | 1.77E-18 | ++ |
| rs11707147 | c | g | 2 | -8.771 | 1.77E-18 | -- |
| rs11720346 | t | c | 2 | 8.771 | 1.77E-18 | ++ |
| rs11720368 | t | g | 2 | 8.771 | 1.77E-18 | ++ |
| rs11720419 | c | g | 2 | -8.771 | 1.77E-18 | -- |
| rs11915624 | a | c | 2 | 8.771 | 1.77E-18 | ++ |
| rs11920057 | a | g | 2 | 8.771 | 1.77E-18 | ++ |
| rs11920108 | t | c | 2 | 8.771 | 1.77E-18 | ++ |
| rs11924446 | a | c | 2 | -8.771 | 1.77E-18 | -- |
| rs11926473 | a | g | 2 | -8.771 | 1.77E-18 | -- |
| rs17243436 | t | c | 2 | 8.771 | 1.77E-18 | ++ |
| rs3849414 | a | t | 2 | 8.771 | 1.77E-18 | ++ |
| rs3889987 | t | g | 2 | 8.771 | 1.77E-18 | ++ |
| rs55961307 | t | g | 2 | -8.771 | 1.77E-18 | -- |
| rs56313346 | t | g | 2 | -8.771 | 1.77E-18 | -- |
| rs73215961 | t | c | 2 | 8.771 | 1.77E-18 | ++ |
| rs73215963 | t | c | 2 | 8.771 | 1.77E-18 | ++ |
| rs73215965 | a | g | 2 | 8.771 | 1.77E-18 | ++ |
| rs73215968 | a | g | 2 | 8.771 | 1.77E-18 | ++ |
| rs73215971 | t | c | 2 | 8.771 | 1.77E-18 | ++ |
| rs28883623 | t | c | 2 | 8.755 | 2.04E-18 | ++ |
| rs55716485 | a | g | 2 | -8.624 | 6.45E-18 | -- |
| rs113630747 | a | c | 2 | 8.539 | 1.36E-17 | ++ |
| rs56202343 | t | c | 2 | 8.539 | 1.36E-17 | ++ |
| rs113346007 | c | g | 2 | 8.523 | 1.56E-17 | ++ |
| rs17183142 | t | g | 2 | 8.523 | 1.56E-17 | ++ |
| rs114539418 | t | c | 2 | 8.488 | 2.10E-17 | ++ |
| rs11716224 | a | c | 2 | -8.458 | 2.71E-17 | -- |
| rs4458369 | a | g | 2 | 8.458 | 2.71E-17 | ++ |
| 3:132091654 | t | c | 2 | -8.365 | 6.00E-17 | -- |
| 3:132092434 | a | t | 2 | 8.365 | 6.00E-17 | ++ |
| rs11717430 | a | g | 2 | 8.31 | 9.55E-17 | ++ |
| rs11718427 | a | g | 2 | 8.31 | 9.55E-17 | ++ |
| rs11926987 | t | c | 2 | 8.31 | 9.55E-17 | ++ |
| rs36015438 | t | c | 2 | 8.31 | 9.55E-17 | ++ |
| rs73215957 | t | c | 2 | -8.31 | 9.55E-17 | -- |
| rs17183254 | a | g | 2 | 8.274 | 1.30E-16 | ++ |
| rs55755772 | a | g | 2 | 8.274 | 1.30E-16 | ++ |
| rs55797446 | t | c | 2 | 8.274 | 1.30E-16 | ++ |
| rs6791064 | t | c | 2 | 8.274 | 1.30E-16 | ++ |
| rs73215949 | a | g | 2 | -8.274 | 1.30E-16 | -- |
| rs73215952 | t | c | 2 | -8.274 | 1.30E-16 | -- |
| rs111913500 | t | g | 2 | 8.12 | 4.65E-16 | ++ |
| rs11714679 | a | g | 2 | -8.12 | 4.65E-16 | -- |
| rs56030168 | a | g | 2 | -8.12 | 4.65E-16 | -- |
| rs61792784 | t | c | 2 | -7.964 | 1.66E-15 | -- |
| rs112950243 | t | c | 2 | 7.936 | 2.08E-15 | ++ |
| rs1157327 | t | g | 2 | 7.936 | 2.08E-15 | ++ |
| rs11708147 | t | c | 2 | -7.936 | 2.08E-15 | -- |
| rs11709247 | a | g | 2 | -7.936 | 2.08E-15 | -- |
| rs11711449 | a | t | 2 | -7.936 | 2.08E-15 | -- |
| rs11711453 | a | g | 2 | -7.936 | 2.08E-15 | -- |
| rs11713275 | t | c | 2 | 7.936 | 2.08E-15 | ++ |
| rs11713782 | t | c | 2 | 7.936 | 2.08E-15 | ++ |
| rs11715301 | t | c | 2 | 7.936 | 2.08E-15 | ++ |
| rs11716689 | t | c | 2 | 7.936 | 2.08E-15 | ++ |
| rs11716692 | t | c | 2 | 7.936 | 2.08E-15 | ++ |
| rs11719814 | t | c | 2 | -7.936 | 2.08E-15 | -- |
| rs16839064 | a | g | 2 | 7.936 | 2.08E-15 | ++ |
| rs2369602 | a | g | 2 | -7.936 | 2.08E-15 | -- |
| rs3905934 | a | g | 2 | 7.936 | 2.08E-15 | ++ |
| rs4101004 | a | c | 2 | -7.936 | 2.08E-15 | -- |
| rs4125177 | c | g | 2 | 7.936 | 2.08E-15 | ++ |
| rs4324499 | t | g | 2 | -7.936 | 2.08E-15 | -- |
| rs56285607 | t | g | 2 | 7.936 | 2.08E-15 | ++ |
| rs56332796 | a | g | 2 | 7.936 | 2.08E-15 | ++ |
| rs59047788 | c | g | 2 | 7.936 | 2.08E-15 | ++ |
| rs59241378 | a | g | 2 | -7.936 | 2.08E-15 | -- |
| rs60561317 | t | g | 2 | 7.936 | 2.08E-15 | ++ |
| rs61792838 | a | g | 2 | -7.936 | 2.08E-15 | -- |
| rs66686529 | a | c | 2 | 7.936 | 2.08E-15 | ++ |
| rs6774168 | a | g | 2 | -7.936 | 2.08E-15 | -- |
| rs6793911 | a | g | 2 | 7.936 | 2.08E-15 | ++ |
| rs6796421 | t | g | 2 | 7.936 | 2.08E-15 | ++ |
| rs6796859 | a | g | 2 | 7.936 | 2.08E-15 | ++ |
| rs6799039 | t | c | 2 | -7.936 | 2.08E-15 | -- |
| rs7620295 | t | c | 2 | 7.936 | 2.08E-15 | ++ |
| rs7621023 | a | g | 2 | -7.936 | 2.08E-15 | -- |
| rs7623271 | a | g | 2 | 7.936 | 2.08E-15 | ++ |
| rs7642300 | a | t | 2 | -7.936 | 2.08E-15 | -- |
| rs4337674 | a | g | 2 | 7.931 | 2.17E-15 | ++ |
| rs61793786 | t | g | 2 | 7.843 | 4.38E-15 | ++ |
| rs34864614 | a | g | 2 | -7.714 | 1.22E-14 | -- |
| rs10935010 | c | g | 2 | 7.172 | 7.39E-13 | ++ |
| rs11719148 | t | c | 2 | 7.077 | 1.47E-12 | ++ |
| rs11719169 | a | g | 2 | 7.077 | 1.47E-12 | ++ |
| rs11916003 | t | c | 2 | -7.077 | 1.47E-12 | -- |
| rs11918232 | a | t | 2 | 7.077 | 1.47E-12 | ++ |
| rs11918882 | t | c | 2 | 7.077 | 1.47E-12 | ++ |
| rs11924491 | a | g | 2 | -7.077 | 1.47E-12 | -- |
| rs11927422 | t | c | 2 | 7.077 | 1.47E-12 | ++ |
| rs16839164 | a | c | 2 | -7.077 | 1.47E-12 | -- |
| rs16839166 | a | t | 2 | -7.077 | 1.47E-12 | -- |
| rs16839170 | a | g | 2 | -7.077 | 1.47E-12 | -- |
| rs2124506 | a | g | 2 | -7.077 | 1.47E-12 | -- |
| rs2168436 | t | c | 2 | -7.077 | 1.47E-12 | -- |
| rs2168437 | a | g | 2 | 7.077 | 1.47E-12 | ++ |
| rs3849413 | a | c | 2 | -7.077 | 1.47E-12 | -- |
| rs4017838 | a | g | 2 | 7.077 | 1.47E-12 | ++ |
| rs4141657 | t | c | 2 | -7.077 | 1.47E-12 | -- |
| rs66507817 | t | c | 2 | 7.077 | 1.47E-12 | ++ |
| rs67408007 | a | g | 2 | -7.077 | 1.47E-12 | -- |
| rs6797900 | a | g | 2 | 7.077 | 1.47E-12 | ++ |
| rs6801391 | a | g | 2 | 7.077 | 1.47E-12 | ++ |
| rs6810217 | t | c | 2 | -7.077 | 1.47E-12 | -- |
| rs72628552 | a | g | 2 | -7.077 | 1.47E-12 | -- |
| rs73215970 | t | c | 2 | -7.077 | 1.47E-12 | -- |
| rs73215972 | t | g | 2 | -7.077 | 1.47E-12 | -- |
| rs11920016 | a | g | 2 | 7.07 | 1.55E-12 | ++ |
| rs10935007 | a | g | 2 | 7.048 | 1.82E-12 | ++ |
| rs11921934 | a | g | 2 | -7.048 | 1.82E-12 | -- |
| rs11929536 | t | c | 2 | 7.048 | 1.82E-12 | ++ |
| rs11929663 | a | g | 2 | 7.048 | 1.82E-12 | ++ |
| rs2124503 | t | c | 2 | -7.048 | 1.82E-12 | -- |
| rs10935008 | t | c | 2 | 7.001 | 2.54E-12 | ++ |
| rs10935009 | a | g | 2 | -7.001 | 2.54E-12 | -- |
| rs12633704 | t | c | 2 | -7.001 | 2.54E-12 | -- |
| rs1500113 | a | g | 2 | -7.001 | 2.54E-12 | -- |
| rs55796117 | a | g | 2 | -7.001 | 2.54E-12 | -- |
| rs67529908 | t | c | 2 | -7.001 | 2.54E-12 | -- |
| rs2310232 | a | g | 2 | -6.986 | 2.83E-12 | -- |
| rs6766469 | a | g | 2 | -6.745 | 1.53E-11 | -- |
| rs73212001 | t | c | 2 | 6.676 | 2.46E-11 | ++ |
| rs10934997 | a | g | 2 | 6.63 | 3.35E-11 | ++ |
| rs73211995 | a | t | 2 | -6.63 | 3.35E-11 | -- |
| rs7630553 | a | g | 2 | 6.63 | 3.35E-11 | ++ |
| rs7632855 | c | g | 2 | -6.63 | 3.35E-11 | -- |
| rs56212843 | c | g | 2 | 6.626 | 3.45E-11 | ++ |
| rs56859075 | a | g | 2 | -6.626 | 3.45E-11 | -- |
| rs11714498 | a | c | 2 | 6.603 | 4.02E-11 | ++ |
| rs56181133 | a | g | 2 | 6.599 | 4.15E-11 | ++ |
| rs73211998 | a | t | 2 | -6.599 | 4.15E-11 | -- |
| rs73213807 | t | c | 2 | 6.599 | 4.15E-11 | ++ |
| rs9847487 | t | c | 2 | 6.593 | 4.30E-11 | ++ |
| rs73215956 | t | c | 2 | -6.589 | 4.43E-11 | -- |
| rs73211991 | a | g | 2 | 6.588 | 4.46E-11 | ++ |
| rs73211996 | a | g | 2 | 6.588 | 4.46E-11 | ++ |
| rs4854894 | a | g | 2 | -6.584 | 4.57E-11 | -- |
| rs3931393 | a | g | 2 | 6.577 | 4.80E-11 | ++ |
| rs73215958 | a | g | 2 | -6.492 | 8.47E-11 | -- |
| rs3905051 | a | c | 2 | -6.441 | 1.19E-10 | -- |
| rs66497351 | t | g | 2 | -6.441 | 1.19E-10 | -- |
| rs6793714 | c | g | 2 | -6.441 | 1.19E-10 | -- |
| rs12374114 | a | c | 2 | -6.388 | 1.68E-10 | -- |
| rs7622003 | a | g | 2 | -6.388 | 1.68E-10 | -- |
| rs7622210 | a | g | 2 | -6.388 | 1.68E-10 | -- |
| rs12633111 | c | g | 2 | -6.374 | 1.84E-10 | -- |
| rs12633125 | a | g | 2 | 6.374 | 1.84E-10 | ++ |
| rs55751856 | c | g | 2 | 6.345 | 2.22E-10 | ++ |
| rs7633760 | a | t | 2 | 6.345 | 2.22E-10 | ++ |
| rs11718382 | a | g | 2 | -6.344 | 2.24E-10 | -- |
| 3:131934872 | a | g | 2 | 6.322 | 2.59E-10 | ++ |
| rs11709606 | a | g | 2 | 6.322 | 2.59E-10 | ++ |
| rs11713348 | t | c | 2 | -6.322 | 2.59E-10 | -- |
| rs35832681 | a | g | 2 | -6.322 | 2.59E-10 | -- |
| rs73211940 | a | g | 2 | 6.322 | 2.59E-10 | ++ |
| rs12639263 | a | g | 2 | 6.31 | 2.78E-10 | ++ |
| rs35420514 | t | g | 2 | -6.31 | 2.78E-10 | -- |
| rs3736565 | t | c | 2 | 6.31 | 2.78E-10 | ++ |
| rs55825707 | a | g | 2 | 6.31 | 2.78E-10 | ++ |
| rs73211962 | a | g | 2 | 6.31 | 2.78E-10 | ++ |
| rs73211969 | a | c | 2 | 6.31 | 2.78E-10 | ++ |
| rs73211970 | a | t | 2 | 6.31 | 2.78E-10 | ++ |
| rs73211976 | t | c | 2 | -6.31 | 2.78E-10 | -- |
| rs73211977 | a | g | 2 | 6.31 | 2.78E-10 | ++ |
| rs7626343 | a | g | 2 | 6.308 | 2.83E-10 | ++ |
| rs56212149 | a | g | 2 | 6.299 | 3.01E-10 | ++ |
| rs56257449 | c | g | 2 | -6.299 | 3.01E-10 | -- |
| rs73211931 | a | c | 2 | -6.299 | 3.01E-10 | -- |
| rs73211934 | t | c | 2 | 6.299 | 3.01E-10 | ++ |
| rs11720059 | a | g | 2 | 6.29 | 3.18E-10 | ++ |
| rs12631837 | t | c | 2 | 6.29 | 3.18E-10 | ++ |
| rs6781382 | a | t | 2 | -6.29 | 3.18E-10 | -- |
| rs6795246 | a | g | 2 | 6.29 | 3.18E-10 | ++ |
| rs73210202 | t | c | 2 | 6.29 | 3.18E-10 | ++ |
| rs73211906 | a | g | 2 | -6.29 | 3.18E-10 | -- |
| rs73211907 | t | c | 2 | 6.29 | 3.18E-10 | ++ |
| rs73211913 | a | g | 2 | 6.29 | 3.18E-10 | ++ |
| rs73211916 | a | t | 2 | 6.29 | 3.18E-10 | ++ |
| rs73211918 | c | g | 2 | 6.29 | 3.18E-10 | ++ |
| rs73211919 | a | g | 2 | 6.29 | 3.18E-10 | ++ |
| rs10934990 | a | g | 2 | 6.276 | 3.48E-10 | ++ |
| rs12636272 | t | c | 2 | 6.276 | 3.48E-10 | ++ |
| rs13059030 | t | c | 2 | -6.276 | 3.48E-10 | -- |
| rs73210196 | a | t | 2 | -6.276 | 3.48E-10 | -- |
| rs56284353 | a | g | 2 | -6.251 | 4.08E-10 | -- |
| rs62410179 | t | c | 2 | 6.237 | 4.45E-10 | ++ |
| rs112926901 | a | g | 2 | 6.207 | 5.41E-10 | ++ |
| rs11709259 | a | g | 2 | 6.207 | 5.41E-10 | ++ |
| rs11709304 | t | c | 2 | 6.207 | 5.41E-10 | ++ |
| rs11714139 | a | g | 2 | 6.207 | 5.41E-10 | ++ |
| rs55910146 | t | c | 2 | 6.207 | 5.41E-10 | ++ |
| rs55931426 | t | c | 2 | -6.207 | 5.41E-10 | -- |
| rs56226080 | c | g | 2 | 6.207 | 5.41E-10 | ++ |
| rs6774580 | a | g | 2 | 6.207 | 5.41E-10 | ++ |
| rs6774876 | a | g | 2 | 6.207 | 5.41E-10 | ++ |
| rs6787261 | t | g | 2 | -6.207 | 5.41E-10 | -- |
| rs73211951 | a | g | 2 | -6.207 | 5.41E-10 | -- |
| rs73211952 | t | c | 2 | -6.207 | 5.41E-10 | -- |
| rs75570634 | a | g | 2 | -6.207 | 5.41E-10 | -- |
| rs76774449 | a | g | 2 | -6.207 | 5.41E-10 | -- |
| rs11720529 | a | g | 2 | -6.195 | 5.82E-10 | -- |
| rs12632159 | t | c | 2 | 6.195 | 5.82E-10 | ++ |
| rs12635861 | a | t | 2 | 6.195 | 5.82E-10 | ++ |
| rs12635862 | a | t | 2 | 6.195 | 5.82E-10 | ++ |
| rs56142574 | t | c | 2 | -6.195 | 5.82E-10 | -- |
| rs56330452 | t | c | 2 | 6.195 | 5.82E-10 | ++ |
| rs73211955 | a | g | 2 | -6.195 | 5.82E-10 | -- |
| rs73211956 | c | g | 2 | -6.195 | 5.82E-10 | -- |
| rs73211958 | a | c | 2 | 6.195 | 5.82E-10 | ++ |
| 6:149198164 | t | c | 2 | -6.169 | 6.86E-10 | -- |
| rs115520762 | t | c | 2 | -5.93 | 3.02E-09 | -- |
| 20:13579060 | a | g | 2 | 5.778 | 7.56E-09 | ++ |
| 20:13598800 | t | g | 2 | 5.778 | 7.56E-09 | ++ |
| rs2180570 | a | c | 2 | 5.778 | 7.56E-09 | ++ |
| rs6042234 | a | g | 2 | -5.778 | 7.56E-09 | -- |
| rs6042241 | t | c | 2 | -5.778 | 7.56E-09 | -- |
| rs6042242 | t | c | 2 | 5.778 | 7.56E-09 | ++ |
| rs6042248 | a | g | 2 | 5.778 | 7.56E-09 | ++ |
| rs6042249 | c | g | 2 | 5.778 | 7.56E-09 | ++ |
| rs6109972 | a | g | 2 | -5.778 | 7.56E-09 | -- |
| rs6033802 | a | g | 2 | -5.764 | 8.22E-09 | -- |
| rs56192118 | a | g | 2 | 5.677 | 1.37E-08 | ++ |
| rs1100396 | a | g | 2 | -5.57 | 2.55E-08 | -- |
| rs11713322 | a | g | 2 | 5.57 | 2.55E-08 | ++ |
| rs16839055 | t | c | 2 | 5.57 | 2.55E-08 | ++ |
| rs2369603 | c | g | 2 | 5.57 | 2.55E-08 | ++ |
| rs14192 | a | g | 2 | -5.568 | 2.57E-08 | -- |
| rs1804136 | t | g | 2 | 5.568 | 2.57E-08 | ++ |
| rs1042330 | a | g | 2 | 5.518 | 3.43E-08 | ++ |
| rs17182812 | t | c | 2 | 5.518 | 3.43E-08 | ++ |
| rs2071505 | a | g | 2 | -5.518 | 3.43E-08 | -- |
| rs3853148 | a | c | 2 | -5.518 | 3.43E-08 | -- |
| rs56302701 | a | g | 2 | -5.518 | 3.43E-08 | -- |
| rs56358880 | t | c | 2 | -5.518 | 3.43E-08 | -- |
| rs10490865 | t | c | 2 | 5.515 | 3.48E-08 | ++ |
| rs11705904 | a | g | 2 | 5.515 | 3.48E-08 | ++ |
| rs17182426 | c | g | 2 | -5.515 | 3.48E-08 | -- |
| rs17182693 | a | g | 2 | -5.515 | 3.48E-08 | -- |
| rs17182707 | a | g | 2 | 5.515 | 3.48E-08 | ++ |
| rs17242260 | c | g | 2 | -5.515 | 3.48E-08 | -- |
| rs17831454 | t | g | 2 | -5.515 | 3.48E-08 | -- |
| rs2228402 | t | c | 2 | 5.515 | 3.48E-08 | ++ |
| rs2290540 | a | g | 2 | -5.515 | 3.48E-08 | -- |
| rs2290541 | a | t | 2 | -5.515 | 3.48E-08 | -- |
| rs2290542 | a | g | 2 | 5.515 | 3.48E-08 | ++ |
| rs34968923 | t | c | 2 | -5.515 | 3.48E-08 | -- |
| rs3749268 | t | c | 2 | -5.515 | 3.48E-08 | -- |
| rs3749269 | t | c | 2 | -5.515 | 3.48E-08 | -- |
| rs3804633 | t | g | 2 | 5.515 | 3.48E-08 | ++ |
| rs56064836 | a | g | 2 | 5.515 | 3.48E-08 | ++ |
| rs57225297 | t | c | 2 | 5.515 | 3.48E-08 | ++ |
| rs58613858 | a | g | 2 | 5.515 | 3.48E-08 | ++ |
| rs59946366 | t | g | 2 | -5.515 | 3.48E-08 | -- |
| rs62291051 | a | g | 2 | 5.515 | 3.48E-08 | ++ |
| rs9790120 | t | c | 2 | 5.515 | 3.48E-08 | ++ |
| rs9790122 | a | g | 2 | 5.515 | 3.48E-08 | ++ |
| rs9790214 | t | g | 2 | -5.515 | 3.48E-08 | -- |
| rs9790218 | a | g | 2 | -5.515 | 3.48E-08 | -- |
| rs1142139 | a | c | 2 | -5.497 | 3.87E-08 | -- |
| rs1142140 | a | c | 2 | -5.497 | 3.87E-08 | -- |
| rs11706447 | t | c | 2 | 5.497 | 3.87E-08 | ++ |
| rs11707236 | t | c | 2 | 5.497 | 3.87E-08 | ++ |
| rs11711309 | t | c | 2 | -5.497 | 3.87E-08 | -- |
| rs4257547 | c | g | 2 | -5.497 | 3.87E-08 | -- |
| rs10935003 | c | g | 2 | -5.494 | 3.93E-08 | -- |
| rs11917521 | t | c | 2 | -5.494 | 3.93E-08 | -- |
| rs11928839 | a | c | 2 | 5.494 | 3.93E-08 | ++ |
| rs11928909 | t | c | 2 | 5.494 | 3.93E-08 | ++ |
| rs11928966 | c | g | 2 | 5.494 | 3.93E-08 | ++ |
| rs55870712 | a | g | 2 | 5.494 | 3.93E-08 | ++ |
| rs17241722 | t | c | 2 | -5.43 | 5.64E-08 | -- |
| rs17831335 | a | g | 2 | -5.43 | 5.64E-08 | -- |
