# Supplementary figures and images for: Variants in ACPP are associated with cerebrospinal fluid Prostatic Acid Phosphatase levels
Source: BMC Genomics. 2016 Jun 29;17(Suppl 3):439. doi: 10.1186/s12864-016-2787-y (PMC4943489; doi:10.1186/s12864-016-2787-y)

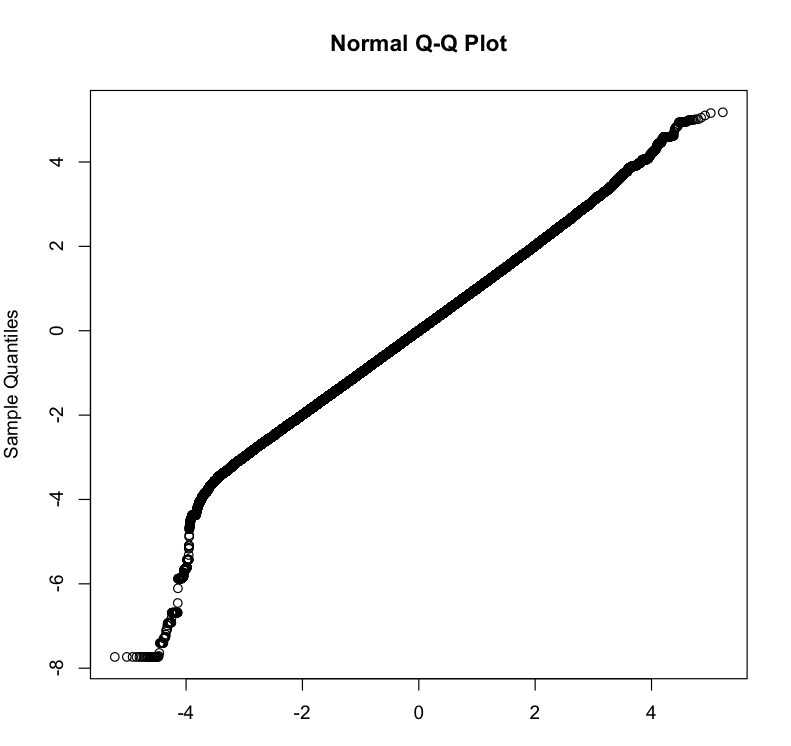

Supplement: Additional file 9: — File contains a Q-Q plot of the CSF in the ADNI samples. (DOCX 54 kb) [file 12864_2016_2787_MOESM9_ESM.docx]

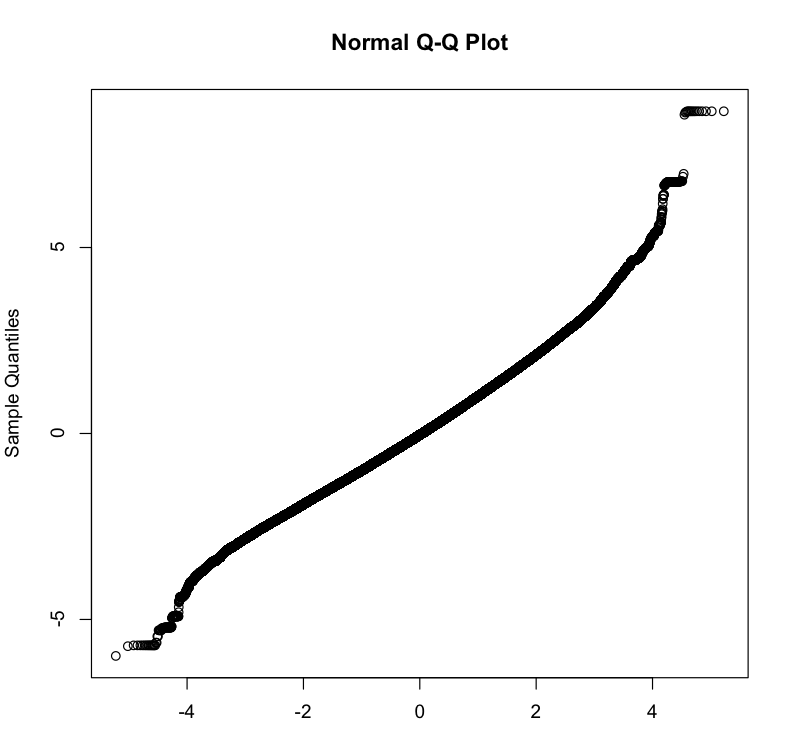

Supplement: Additional file 10: — File contains a Q-Q plot of the CSF in the Knight ADRC samples. (DOCX 52 kb) [file 12864_2016_2787_MOESM10_ESM.docx]
